# Examination of the diagnostic accuracy of sepsis using procalcitonin, presepsin and CD64 for patients with or without acute kidney injury

**DOI:** 10.1186/cc14144

**Published:** 2015-03-16

**Authors:** Y Nakamura, H Ishikura, J Tanaka, T Nishida, M Mizunuma, D Ohta, N Matsumoto, A Murai

**Affiliations:** 1 Fukuoka University Hospital, Fukuoka, Japan

## Introduction

At the moment, we have few reports about the diagnostic accuracy of procalcitonin (PCT), presepsin (P-SEP) and CD64 as a diagnostic marker of sepsis for patients with acute kidney injury (AKI). This study aimed to clarify which is a more useful diagnostic biomarker for sepsis using PCT, P-SEP and CD64 with or without AKI in ICU patients.

## Methods

This study was a single-center observational retrospective study. Blood samples were collected from 334 patients admitted to our ICU between April 2013 and March 2014. Then, we classified the patients with or without AKI. In this study, we adopted RIFLE criteria for AKI diagnosis. After that, the patients in each group were classified into the sepsis group and the nonsepsis group. We measured PCT, P-SEP and CD64 levels at the time of ICU admission and subsequently investigated the diagnostic accuracy of these biomarkers for detecting sepsis.

## Results

In this study we met 225 patients with non-AKI and 109 patients with AKI. We conducted ROC analysis for diagnosing sepsis. In non-AKI patients, the AUC of PCT, P-SEP and CD64 were 0.904 (95% CI: 0.824 to 0.950), 0.892 (95% CI: 0.794 to 0.947) and 0.917 (95% CI: 0.842 to 0.958), respectively. In AKI patients, the AUC were 0.933 (95% CI: 0.859 to 0.970), 0.755 (95% CI: 0.642 to 0.840) and 0.905 (95% CI: 0.803 to 0.957), respectively.

## Conclusion

CD64 and PCT were a useful biomarker for detecting sepsis for ICU patients with AKI.

